# Febrile sepsis: first report of human disease due to *Paenibacillus silvae*

**DOI:** 10.1099/acmi.0.000580.v3

**Published:** 2023-06-23

**Authors:** Ilaria Dalla Vecchia, Daniele Fasan, Manuela Pegoraro, Paolo Benedetti

**Affiliations:** ^1^​ Unità Operativa di Malattie Infettive, Ospedale San Bortolo, Vicenza, Italy; ^2^​ Laboratorio di Microbiologia, Ospedale San Bortolo, Vicenza, Italy

**Keywords:** *Paenibacillus silvae*, febrile sepsis

## Abstract

**Introduction.:**

*
Paenibacillus* species are saprophytes widely distributed in nature and rarely associated with overt human infection. Most cases have been described in people with important comorbidities and/or immunodepression. We report here what is, to the best of our knowledge, the first documented case of human disease due to *Paenibacillus silvae *, so far considered an exclusively environmental micro-organism.

**Case presentation.:**

A 57-year-old female patient was referred to our Unit after a 2 month history of remittent fever. Upon admission, a septic state and bacteraemia were revealed; *P. sylvae* was identified by 16S rRNA gene amplification and sequencing with matrix-assisted laser desorption/ionization-time of flight MS. The patient became afebrile after 9 days of antibiotic treatment and was completely cured after a 2 week regimen with intravenous amoxicillin-clavulanate plus oral doxycycline.

**Conclusion.:**

The patient did not report any previous episode of infection. Most of the well-known risk factors to *Paenibacillus* bacteraemia, i.e. invasive procedures, use of intravenous drugs and foreign bodies, could be excluded, although her immune system was probably impaired due to obesity and heavy smoking. We suggest that the isolation of bacteria belonging to the genus *Paenibacillus* should not be disregarded, since there is accumulating evidence that these organisms may cause disease even in immunocompetent subjects.

## Data Summary

No new data were generated in this study. References are reported with DOI or PMID/PMCID in the only case where a DOI was not available.

## Introduction

The genus *Paenibacillus* comprises rod-shaped Gram-positive or Gram-variable, aerobic or facultatively anaerobic bacteria, which form endospores in the presence of oxygen. Formerly included in the genus *Bacillus *, it was categorized in 1993 as a novel genus [1], so far containing 294 recognized species [2]. *Paenibacillus* species are saprophytes widely distributed in nature, particularly in soil, from where they are spread in dust, in water, and on materials of plant and animal origin [3]. They are known to cause spoilage of dairy products [4] and can be pathogens to honeybees and other invertebrates [5]. Despite their widespread distribution, the majority of species are rarely associated with overt human infection. Such associated species are more frequently isolated from elderly patients whose immune systems are generally weak, or they are considered to be culture contaminants. However, cases of bacteraemia [6–11], endocarditis [12, 13], neonatal meningitis [14–16], pneumopathy [17, 18], urinary infection [19], skin infection [20, 21], arthritis [22], post-traumatic osteomyelitis [23], ocular disease [24] and prosthetic infection [25] have been described. In some of the above cases, especially if the patient was immunocompetent, bacteraemia presented as a relatively benign condition and antimicrobial therapy was started after specific sensitivity results.

*Paenibacillus* spp. isolated from humans have demonstrated a variety of strain-dependent drug resistances, including resistances to norfloxacin, clindamycin, ampicillin and ticarcillin/clavulanic acid. Successful drug treatments have included cefotaxime [19] and combination therapies with either amikacin and piperacillin/tazobactam (followed by oral levofloxacin) [7] or ceftriaxone plus vancomycin [25].

*Paenibacillus silvae* (belonging to the *Paenibacillus amylolyticus* group) [26] was first isolated in 2017 from a tropical rainforest soil [27]. The original strain exhibited rod-shaped, immotile cells, positive for catalase, nitrate reduction and the methyl red test but negative for oxidase and the Voges-Proskauer reaction. Growth occurred between pH 5.0 and 11.0, between 10 and 53 °C, and in 0–4 % (w/v) NaCl. It tested as being susceptible to acheomycin, ampicillin, chloromycetin, erythromycin, kanamycin, gentamicin, nalidixic acid, neomycin, novobiocin and rifampicin.

We report here what is, to the best of our knowledge, the first documented case of human disease due to *P. silvae *, so far considered an exclusively environmental micro-organism.

## Case presentation

A 57-year-old woman with a history of psychosis, obesity and dyslipidaemia was referred to our Unit. She admitted to smoking 120 packets of cigarettes a year but denied alcohol misuse. Her medical and surgical histories were otherwise unremarkable. For many years she had been voluntarily providing foster care and reported exposures to potentially contaminated water. The patient sought medical advice after a 2 month history of fever, which displayed peaks at 39 °C with chills and exhibited remitting courses lasting on average 4–6 days, followed by brief (1–2 days) afebrile spells. During this time, she had received two courses of antibiotics, both of 7 day duration (the first with oral amoxicillin, started 5 days after symptom onset; and the second, 3 weeks later, with oral levofloxacin). The patient was transiently responsive to both courses, displaying abatement of temperature for 5–6 days after each treatment.

Upon admission, the patient was febrile (38.5 °C) and slightly hypotensive, with blood pressure of 100/70 mmHg. She displayed reduced diaphragmatic excursion due to morbid obesity (weight 108 kg, height 155 cm) but chest and cardiac auscultation, as well as abdominal and neurological examinations, were normal. Pertinent laboratory results included a white blood cell count of 8400 cells µl^−1^ with normal neutrophils, a C-reactive protein (CRP) of 4.3 mg dl^−1^, procalcitonin (PCT) of 1.05 µg l^−1^, and normal kidney and liver examinations. Reverse transcriptase PCR testing for SARS-CoV-2 was negative. A chest X-ray revealed mild broncho-vascular markings in the left pulmonary base.

Given the patient's stable clinical condition, no antibiotics were administered pending the results of peripheral blood cultures collected upon admission, which further signalled positivity for Gram-positive bacilli. The micro-organism was identified as *Paenibacillus* spp. by 16S rRNA gene amplification and sequencing with matrix-assisted laser desorption/ionization-time of flight (MALDI-TOF) MS. MS analysis was performed on a mass spectrometer (Bruker Reflex III time-of flight spectrometer; Bruker Daltonics Inc); the cut-offs of identification scores were categorized into ≥2.000, 1.700–1.999 and <1.700 according to the manufacturer’s recommendation. A score of ≥2.000 represented reliable identification at the species level, and <1.700 was interpreted as no identification. Our strain was identified as representing *P. silvae* with a score >2.01. It was fully sensitive to ampicillin, gentamicin, ciprofloxacin, linezolid, rifampicin, vancomycin and tetracycline, but resistant to clindamycin.

Based upon sensitivity testing, the patient was started on intravenous amoxicillin/clavulanate 2.2 g t.i.d. plus oral doxycycline 100 mg b.i.d. for 2 weeks. Her fever abated at day 9 after treatment initiation and the patient remained afebrile until being discharged. CRP and PCT returned to normal levels 10 days after antibiotic commencement. Both trans-thoracic and trans-oesophageal echocardiograms failed to reveal endocardial vegetations. Amongst the other investigations performed, orthopantomography showed partial edentulism and periapical granuloma of the left lower molar, and colonoscopy (performed as required by an internal diagnostic protocol for febrile disease) detected a tubular adenoma with low-grade dysplasia at the right colonic flexure, which was endoscopically excised. Given the patient's history of long-lasting febrile disease with transient response to antibiotics, a total-body positron emission tomography/computed tomography examination was also done to rule out endocarditis and/or alternative septic foci, without revealing significant hypermetabolic focalities.

The patient was asymptomatic and displayed normal serum inflammation markers when discharged at completion of antimicrobial treatment, with no further follow-up available.

## Discussion

The patient did not remember any previous episode of infection. Most of the well-known risk factors to *Paenibacillus* bacteraemia, i.e. invasive procedures [6, 7, 23], use of intravenous drugs [9, 10], major immunosuppressive conditions [7, 17, 21, 24] and foreign bodies [12, 25], could not be identified. However, her immunity was probably impaired due to obesity and heavy smoking. Whilst in hospital, two potential portals of infection were detected, i.e. a periapical granuloma and a colonic tubular adenoma.

As in the majority of cases reported in apparently immunocompetent subjects, the clinical presentation of sepsis was relatively mild, allowing sensitivity testing results to be made before starting antibiotics; the final outcome was favourable. In fact, pre-existing immunosuppressive comorbidities are known to have an adverse impact on the clinical course of *Paenibacillus* infection [10, 18, 24] and, interestingly, the reported paediatric cases of *Paenibacillus* spp. infections to date have been mainly in infants born prematurely who developed severe central nervous system infections leading to poor outcomes [14, 16]. Recent reports from developing countries have also revealed that *Paenibacillus* is the dominant pathogen in infants with post-infective hydrocephalus following a history of prior neonatal sepsis, causing brain ventriculitis resulting in calcified loculations and abscesses [28–30]. In such a context, *Cytomegalovirus* co-infection was frequently present [29, 30], but the analysis of cerebrospinal fluid failed to detect any functional protein associated with a response to *Cytomegalovirus* whilst, only in *Paenibacillus *-positive children, it revealed neutrophil-mediated inflammation, negative regulation of proteolysis and peptidase activity, and modulation of extracellular matrix and structure [30].

The ability of *Paenibacillus* species to produce biofilm, enterotoxins and proteases has been well established [31]; in particular, thiaminases I and II allow the microorganisms to adapt to living in the presence of thiamine-deficiency, as often occurs in well-known predisposing factors to *Paenibacillus* infection such as alcohol addiction, sickle-cell disease and malnutrition. Therefore, we suggest that the isolation of bacteria belonging to the genus *Paenibacillus* should not be disregarded, since there is accumulating evidence that these organisms may cause disease even in immunocompetent subjects. The use of more accurate identification methods, often requested such as for coagulase-negative staphylococci, *Streptococcus* spp. and bacteria with complex nutritional requirements, should be prompted by clinicians even for Gram-variable bacteria, since the use of current biochemical identification systems may lead to misidentification as other bacteria. Accordingly, it is expected that proper identification of this genus will help clinical physicians to make decisions regarding appropriate treatment and use of antibiotics.
